# Synergistic associations between dietary patterns, lifestyle behaviors and childhood obesity

**DOI:** 10.3389/fnut.2026.1817777

**Published:** 2026-07-06

**Authors:** Bingbing Guo, Lan Jiang, Xinye Jiang

**Affiliations:** Wuxi Maternity and Child Health Hospital, Affiliated Women’s Hospital of Jiangnan University, Wuxi, Jiangsu, China

**Keywords:** children, correlation, dietary behavior, dietary diversity score, growth and development

## Abstract

**Objective:**

To analyze the relationships between diet, lifestyle behaviors and obesity among preschool children aged 3–7 in Wuxi City, providing a scientific basis for implementing a healthy diet and lifestyle.

**Methods:**

A stratified cluster random sampling method was used to select study participants. Self-administered questionnaires were used to collect demographic data. The dietary diversity score (DDS), dietary behaviors, and current lifestyle were analyzed. Children’s growth and development were assessed using BMI-Z scores. Variance analysis and logistic regression were employed to explore the relationships.

**Results:**

A total of 3,727 preschool children were included. The average DDS was 5.07 ± 1.94, with 36.79% of children showing low dietary diversity (DDS ≤ 4). The largest proportion of children (53.13%) had normal BMI-Z score (−1 ≤ BMI-Z ≤ 1). The children were divided into four groups based on BMI-Z scores, and statistically significant differences were observed across age, current height, current weight, birth weight, DDS, parental BMI, and maternal pre-pregnancy weight (*p* < 0.05). Low dietary diversity, nighttime snacking, and prolonged daily screen time (1-2 h or ≥2 h) were positively associated with childhood obesity (*p* < 0.05). After adjusting for factors including children’s age, gender, birth weight, parental BMI, family income, number of family members dining together, family type, and primary caregiver, low dietary diversity remained significantly associated with obesity.

**Conclusion:**

To prevent childhood obesity, more attention should be paid to preschool children with low dietary diversity, frequent nighttime snacking and excessive screen use. Targeted interventions addressing these factors should be considered.

## 1 Introduction

Childhood growth and development covers physical, cognitive and social abilities. With socioeconomic development and changes in lifestyles, shifts in dietary patterns and over-nutrition have resulted in varied prevalence of childhood obesity nationwide. A national survey covering 42,982 Chinese children from 2010 to 2020 found that overweight was common among adolescents, with a continuous increase in obesity rates. Meanwhile, the prevalence of obesity and severe obesity gradually decreased among children aged 2–6 years (1). Overweight and obesity remain prevalent among children and adolescents in China, and obesity-related metabolic diseases are becoming increasingly common (2). As an independent risk factor for multiple chronic diseases, childhood obesity predisposes individuals to early hypertension and atherosclerosis, and raises lifelong risks of type 2 diabetes and persistent obesity (3, 4). Moreover, children who experience early malnutrition followed by excessive weight gain tend to have disrupted metabolic homeostasis, increasing their susceptibility to non-communicable diseases (5). If no effective interventions are implemented, the ongoing overweight and obesity epidemic among Chinese children and adolescents will lead to a loss of 3.3 billion disability-adjusted life years (DALYs). In addition, the associated lifetime economic loss will reach as high as 218 trillion Chinese yuan (approximately 31.6 trillion United States dollars) (6). Clarifying the relationships between diet, lifestyle and pediatric obesity and implementing targeted interventions are therefore critical to promoting long-term population health. Earlier studies mainly focused on the associations between individual nutrients and obesity (7, 8). Currently, dietary patterns and eating behaviors are recognized as key determinants of children’s growth and development, as they reflect the overall impacts of daily food consumption and better interpret the association between diet and chronic diseases (9). The dietary diversity score (DDS) is a validated indicator for evaluating dietary quality and nutritional adequacy (10), and food diversity has been adopted as a core component of healthy diets in many countries (11, 12). The Chinese Dietary Guidelines (2022) also recommends consuming at least 12 kinds of foods per day and 25 kinds per week (12). However, existing studies on dietary diversity and body weight have yielded inconsistent results across populations (13–15). In addition, unhealthy dietary behaviors such as nighttime snacking and excessive consumption of ultra-processed foods may affect children’s weight management. This study aimed to assess preschool children’s growth and development using BMI-Z scores, and explore the correlations of dietary patterns, eating behaviors and lifestyle habits with BMI-Z scores. We further analyzed the association between dietary diversity and growth status in children aged 3–7 years.

## 2 Methods

### 2.1 Participants

A stratified cluster random sampling method was used to select guardians of preschool children enrolled in kindergartens across 8 districts of Wuxi City in 2024. In total, 3,727 guardians from 18 kindergartens were included in this study.

### 2.2 Survey methods and content

The study utilized a self-designed questionnaire based on the multi-indicator cluster survey developed by UNICEF, which has high reliability and validity (16). The questionnaire was distributed online, covering the following areas: Child Demographic Information: Gender, age, height, weight, birth weight. Parental Information: Weight, height, education level, occupation, maternal age at pregnancy, pre-pregnancy weight. Household Information: Monthly per capita income, household size, primary guardian type. The 24-Hour Dietary Recall for Children was used to assess children’s daily diet. In this study, the 24-h dietary recall collected information on children’s usual daily diets as reported by their guardians, excluding unusual days (festivals, sick days and occasional dining out) to minimize bias from atypical dietary intake.

### 2.3 Evaluation indicators and definitions

#### 2.3.1 Dietary diversity

Defined according to the “Guidelines for Measuring Household and Individual Dietary Diversity” by the Food and Agriculture Organization (FAO), categorizing daily food intake into nine groups: cereals, tubers, vegetables, fruits, legumes/nuts, meat, fish, eggs, dairy products. Each group consumed within 24 h received 1 point, with a maximum score of 9. Low dietary diversity was defined as a score ≤4 (17).

#### 2.3.2 BMI-Z score

Used to assess children’s growth and development, calculated using the World Health Organization (WHO) standards (18). A BMI-Z score ≤−1 indicated underweight, −1 < BMI-Z < 1 indicated normal weight, 1 ≤ BMI-Z < 2 indicated overweight, and BMI-Z ≥ 2 indicated obesity (19).

#### 2.3.3 Nighttime snacking

Defined as consuming food within 2 hours before bedtime.

#### 2.3.4 Ultra-processed foods

Included sugary drinks (e.g., soda, milk tea, fruit juices), processed meats (e.g., sausages, bacon), fried foods (e.g., fries, fried chicken), sweets (e.g., candy, ice cream, cakes), and puffed snacks (e.g., chips, biscuits). Daily intake of any of these foods was defined as regular consumption of ultra-processed foods.

#### 2.3.5 Household type

Defined as single-parent households, remarried families, nuclear families, or extended families.

#### 2.3.6 Child lifestyle habits

Included daily sleep duration (including naps), daily screen time, and daily outdoor activity duration.

### 2.4 Quality control

Before conducting the survey, we consulted with pediatricians, kindergarten teachers, and parent representatives to ensure the feasibility and scientific validity of the questionnaire. The questionnaire was designed to include response options for every question and embedded logical validation rules to reduce data entry errors. Two investigators independently reviewed the collected data, and any discrepancies were resolved by a third party to ensure data quality.

### 2.5 Statistical analysis

Statistical analysis was conducted using SPSS 19.0 software. Continuous variables were expressed as mean ± standard deviation (mean ± SD), and categorical variables were expressed as percentages (*n*%). Group comparisons were conducted using analysis of variance (ANOVA), with pairwise comparisons between multiple groups performed using the SNK-q test. Logistic regression was used to examine the associations between dietary diversity, nighttime snacking, ultra-processed food intake, outdoor activity time, screen time, sleep duration and obesity. After adjusting for statistically significant differences and core variables, logistic regression was used to evaluate the impact of low dietary diversity (yes = 1, no = 0) on childhood obesity (yes = 1, no = 0), with statistical significance set at *p* < 0.05.

## 3 Results

### 3.1 General characteristics

A total of 3780 questionnaires were collected, of which 53 were excluded due to missing information, resulting in 3727 valid responses, with an effective rate of 98.60%. The questionnaires covered kindergartens from all districts of Wuxi City, with the children’s ages ranging from 3 to 7 years old, averaging 5.35 ± 0.98 years. There were 1924 boys (51.62%) and 1803 girls (48.38%). The majority of the children (53.13%) had a BMI-Z score between −1 and 1. The average dietary diversity score (DDS) was 5.07 ± 1.94, with 36.79% of children having low dietary diversity (DDS ≤ 4).

### 3.2 Characteristics by BMI-Z group

Based on BMI-Z scores, children were divided into four groups: underweight (BMI-Z < −1), normal weight (−1 ≤ BMI-Z < 1), overweight (1 ≤ BMI-Z < 2), and obese (BMI-Z ≥ 2). Comparisons between the four groups revealed significant differences in age (*F* = 7.936, *p* < 0.001), height (*F* = 9.879, *p* < 0.001), current weight (*F* = 1360.672, *p* < 0.001), birth weight (*F* = 2.645, *p* = 0.036), DDS (*F* = 5.657, *p* < 0.001), maternal BMI (*F* = 34.735, *p* < 0.001), maternal pre-pregnancy weight (*F* = 41.905, *p* < 0.001), and paternal BMI (*F* = 55.017, *p* < 0.001). See Table 1 for details.

**Table 1:** Characteristics of preschool children in different BMI-Z groups.

BMI-Z	*N*	Age	Height	Weight	Birth weight (kg)	DDS	Maternal BMI	Maternal age during pregnancy	Maternal pre-pregnancy weight	Paternal BMI
≤−1	663	5.49 ± 1.00	114.83 ± 8.14	17.87 ± 3.44	4.15 ± 1.74	5.03 ± 1.98	23.91 ± 11.58	29.04 ± 6.98	76.81 ± 25.78	25.32 ± 6.96
−1~1	1980	5.34 ± 0.96	113.46 ± 8.00	20.10 ± 3.69	3.77 ± 1.55	5.18 ± 1.88	24.08 ± 7.76	28.67 ± 4.53	79.87 ± 26.85	25.96 ± 6.34
1~2	408	5.52 ± 1.01	115.42 ± 8.98	23.19 ± 4.34	3.84 ± 1.28	4.98 ± 1.87	24.63 ± 8.41	28.10 ± 3.94	81.50 ± 30.87	25.94 ± 6.71
≥2	676	5.40 ± 1.00	113.85 ± 9.58	33.84 ± 4.64	4.76 ± 1.68	4.84 ± 2.13	28.05 ± 10.38	28.59 ± 4.80	92.18 ± 35.64	29.88 ± 11.20
*F*		7.936	9.879	1360.672	2.645	5.657	34.735	1.848	41.905	55.017
*p*		<0.001	<0.001	<0.001	0.036	0.001	<0.001	0.136	<0.001	<0.001

### 3.3 Associations between dietary/lifestyle behaviors and obesity

Logistic regression analysis was performed to examine the association between various dietary and lifestyle behaviors and obesity. The independent variables included dietary diversity score (using the normal dietary pattern, DDS ≥ 5 group as the reference), nighttime snacking (using the “no” group as the reference), daily consumption of ultra-processed foods (using the “no” group as the reference), daily outdoor activity time (using the >2 h/day group as the reference), daily screen time (using the ≤0.5 h group as the reference), and average daily sleep duration (using the <9 h group as the reference). The dependent variable was obesity (no = 0, yes = 1). After adjusting for age and gender, we found that low dietary diversity (OR = 1.183, 95% CI: 1.101–1.203), nighttime snacking (OR = 1.101, 95% CI: 1.092–1.512), 1-2 h of screen time daily (OR = 1.328, 95% CI: 1.094–1.614), and ≥2 h of daily screen time (OR = 1.522, 95% CI: 1.087–2.130) were positively associated with obesity in preschool children (*p* < 0.05). See Table 2 for details.

**Table 2:** Correlation of dietary behaviors and lifestyle with obesity.

Factors	Male	Female	Total
DDS			
≥5	1.00	1.00	1.00
≤4	1.098 (1.022–1.159)*	1.210 (1.105-1.238)*	1.183 (1.101-1.203)*
Nighttime snacking			
No	1.00	1.00	1.00
Yes	1.092 (1.082–1.101)*	1.108 (1.009-1.332)*	1.101 (1.092-1.512)*
Ultra-processed food			
No	1.00	1.00	1.00
Yes	1.089 (0.895–1.325)	0.896 (0.726–1.107)	0.909 (0.787–1.049)
Outdoor activity time			
>2 h/d	1.00	1.00	1.00
≤2 h/d	0.927 (0.749–1.147)	0.864 (0.683–1.094)	0.904 (0.771–1.060)
Daily screen time			
≤0.5 h	1.00	1.00	1.00
0.5-1 h	0.849 (0.676–1.066)	1.174 (0.917–1.503)	0.981 (0.830–1.161)
1-2 h	1.319 (1.011–1.723)*	1.351 (1.017-1.795)*	1.328 (1.094-1.614)*
≥2 h	1.253 (0.800-1.964)	1.980 (1.195–3.280)*	1.522 (1.087-2.130)*
Average daily sleep time			
<9 h	1.00	1.00	1.00
9–13 h	0.936 (0.689–1.271)	0.722 (0.518–1.006)	0.850 (0.677–1.067)
≥13 h	0.738 (0.368–1.481)	1.844 (0.938–3.624)	1.201 (0.744–1.939)

*p < 0.05; obesity (no = 0, yes = 1) was the dependent variable, adjusted for age and gender.

### 3.4 Logistic regression analysis of dietary patterns and childhood obesity

Logistic regression analysis revealed that low dietary diversity was a significant risk factor for obesity, with a crude OR of 1.216 (95% CI: 1.051–1.406). After adjusting for child-related factors such as age, gender, and birth weight, the OR in Model 1 was 1.192 (95% CI: 1.020–1.392). In Model 2, after further adjusting for maternal and paternal BMI, the OR was 1.195 (95% CI: 1.017–1.404). In Model 3, after additionally adjusting for household income, family dining practices, family type, primary caregiver, screen time and physical activity, the OR was 1.204 (95% CI: 1.017–1.389) (*p* < 0.05). See Table 3 for details.

**Table 3:** Logistic regression analysis of dietary diversity and obesity.

Model	*B*	S.E	Wals	*p*	OR	95% C.I.
Crude OR	0.195	0.074	6.940	0.008	1.216	1.051~1.406
OR model 1	0.176	0.079	4.899	0.027	1.192	1.020~1.392
OR model 2	0.178	0.082	4.681	0.030	1.195	1.017~1.404
OR model 3	0.162	0.091	5.259	0.021	1.204	1.017~1.389

Model 1: adjusted for child-related factors (age, gender, birth weight). Model 2: additionally adjusted for maternal and paternal BMI. Model 3: additionally adjusted for household income, family dining practices, family type, primary caregiver, screen time and physical activity.

## 4 Discussion

This study used BMI-Z scores to assess the nutritional and developmental status of preschool children. It was found that the rates of overweight (1 ≤ BMI-Z < 2) and obesity (BMI-Z ≥ 2) in children were 10.94 and 18.14%, respectively, which were significantly higher than the national prevalence rates of 6.8 and 3.6% for overweight and obesity in children under 6 years old during 2015–2019 (20). With the rapid socioeconomic development and lifestyle changes in China, particularly the increase in nutrition levels and more diverse diets, the growth and development of children have improved. Although the rates of overweight and obesity among Chinese children are still lower than those in developed countries, yet their rising trends are similar to those observed in developed countries. If timely interventions are not implemented, childhood obesity rates could catch up with or even surpass those in developed nations. The development of childhood obesity is influenced by various factors, including genetics, environment, and dietary behaviors. Although genetic factors play an important role in the onset and progression of obesity, genetic factors remain relatively stable throughout the preschool period (ages 3–7). Therefore, the occurrence of overweight and obesity in children at this stage is mainly attributed to changes in the external environment and social factors. Thus, the prevention and control of childhood obesity should primarily focus on environmental, behavioral, and lifestyle factors. The external environmental factors that contribute to childhood obesity include changes in dietary structure (21), reduced outdoor activities, and unhealthy eating behaviors (22). Changes in eating behaviors, such as increased meal frequency, higher intake of ultra-processed foods, and sugary drinks replacing primary energy sources, also play a role. This study primarily explored these factors influencing childhood obesity.

In this study, the average dietary diversity score (DDS) for children aged 3–7 was 5.07 ± 1.94, with 36.79% of children having low dietary diversity (DDS ≤ 4). The DDS quantifies the number and variety of food groups consumed in the diet, serving as an effective indicator of micronutrient intake (23). A higher DDS reflects a greater variety of nutrient intake, which is beneficial for children’s physical development (24). Some international studies have shown that higher DDS is associated with better growth outcomes in children (25, 26) We found that low dietary diversity and nighttime snacking were risk factors for childhood obesity. A single dietary pattern that lacks essential high-quality proteins and vegetables while overemphasizing carbohydrates, starches, and fats lays the foundation for obesity in children. A longitudinal study in the United States found a negative correlation between weight gain and the consumption of fruits and vegetables (27). As household disposable income increases, so does the demand for better-tasting and higher-quality food, beyond merely satisfying hunger. Consequently, the food industry creates and processes food that is lower in nutritional value but more palatable, promoting unhealthy eating habits. Children and adolescents, who generally lack the ability to distinguish between healthy and unhealthy foods, are often influenced by the retail food environment around schools. Studies have shown that the availability of sugary beverages in school stores and the proximity of Western fast food outlets and convenience stores near schools are associated with higher body mass index (BMI) and increased obesity rates (28, 29). In addition, the consumption of ultra-processed foods and various snack products is on the rise in China. A study comparing packaged foods and beverages from 12 countries found that sugar and saturated fat content in China’s packaged foods is among the highest (30). A national survey involving 53,151 children and adolescents in China found that 42% consumed sugary drinks at least twice a week, and a cross-sectional analysis of 203 participants revealed a significant association between high consumption of sugary beverages and an increased likelihood of obesity (31, 32). Several studies have also linked the increased consumption of ultra-processed foods and beverages with higher BMI levels (33). The survey on nighttime snacking also indicated that foods consumed late at night tend to be high in fat and sugar, making them more appealing to children. This leads to increased meal frequency and reduced dietary diversity, both of which contribute to weight gain. In this study, we analyzed the dietary status of children with low dietary diversity. The results showed that their dietary patterns were monotonous, with refined grains, fried foods and sweets -foods high in energy but low in nutritional density -serving as the staple foods, while the intakes of vegetables, fruits, legumes and dairy products were inadequate. A monotonous diet may lead to excessive total energy intake and nutritional imbalance, thus increasing obesity risk (34, 35). This elevated risk is likely linked to limited food diversity, systemic inflammation and gut microbiota dysbiosis, which impair nutrient utilization and induce metabolic dysfunction. Children with excess energy intake accompanied by inadequate nutrient supply commonly suffer from deficiencies in vitamin D, calcium, iron, magnesium and B vitamins, along with imbalanced intake of macronutrients. Diets high in saturated fat and refined carbohydrates exacerbate inflammatory responses and increase the risk of metabolic diseases, whereas plant-based protein, unsaturated fat and dietary fiber help maintain metabolic homeostasis (36–38). We also investigated the types of foods consumed as night snacks among the participants. It was found that children with a low dietary diversity index were more likely to have night snacks, which mainly consisted of sugary beverages, puffed foods and fried products. Frequent night eating, combined with monotonous dietary patterns and shortened sleep duration, collectively leads to excessive gains in body weight and body fat (39). Accumulating evidence indicates that the timing and nutritional composition of food intake act as crucial zeitgebers for the human biological clock. Accordingly, eating at inappropriate time points such as nighttime can disrupt circadian rhythms, and long-term irregular eating may result in a range of adverse health outcomes, including weight gain, obesity and metabolic disorders (40). Notably, this cross-sectional study cannot establish causal direction. Parents of children who are overweight or obese may deliberately limit food varieties and snacks to control their children’s weight, which may lead to lower dietary diversity among obese children (reverse causality). Future longitudinal cohort studies are required to monitor dynamic changes in diet, lifestyle and weight status, so as to clarify the causal sequence between dietary diversity and childhood obesity.

We also found that excessive screen time was another significant risk factor for childhood obesity. Prolonged screen time leads to sedentary behavior and reduced physical activity, both of which are key contributors to obesity. Several studies have confirmed that insufficient physical activity and prolonged sedentary behavior are common among children and adolescents. Although some studies did not find a direct correlation between moderate or high levels of physical activity and lower body weight (41, 42), observational studies suggest that children who engage in more physical activity are less likely to be overweight or obese compared to those with insufficient physical activity (43). Additionally, a decrease in walking to school has been identified as a predictor of childhood obesity. Thus, we believe that a lack of physical activity, combined with extended screen time, likely increases the risk of obesity in children (44).

This study comprehensively collected information on children, their mothers and families, and explored factors associated with the growth and development of preschool children from multiple perspectives including children’s dietary patterns, behavioral and living habits. This comprehensive analytical approach lays a scientific foundation for future research on child growth and development. Nevertheless, several limitations of this study should be acknowledged. First, the dietary diversity score (DDS) was calculated based on a single 24-h dietary recall, which fails to fully reflect children’s long-term habitual dietary intake. A single-day dietary record is susceptible to random variations, which may cause minor misclassification of dietary diversity. In future studies, three consecutive days of dietary recall combined with food frequency questionnaires will be used to evaluate long-term dietary habits. Second, the sample size was relatively limited. This research was conducted in a single city in China, so the findings may not be generalizable to the whole country due to substantial regional disparities. Although stratified cluster sampling was adopted to ensure the representativeness of the samples and reduce survey costs, potential selection bias still existed. Third, as a cross-sectional study, this research can only demonstrate correlations rather than definite causal relationships between the investigated factors and children’s growth and development. In addition, the online questionnaire completed by children’s guardians may introduce potential response bias. To minimize such bias, we adopted stratified cluster sampling and held multiple meetings to verify the feasibility and scientificity of the survey. Logical validation functions were embedded in the questionnaire to prevent data entry errors. Once obvious errors or missing data were identified, we promptly contacted guardians via class teachers to supplement relevant information and guarantee data quality. Considering the characteristics of preschool children, having their guardians complete the online questionnaires on their behalf effectively reduced response bias and improved survey efficiency and data accuracy.

Childhood obesity is recognized as a disease in itself and is closely linked to multiple non-communicable diseases. It is also a major risk factor for adult obesity, placing a significant economic burden on society. Proactive dietary and physical activity interventions have been shown to yield favorable cost–benefit outcomes (45). Therefore, strategies for the early prevention and control of childhood obesity should begin at a young age, including educating and managing maternal weight during pregnancy to prevent excessive weight gain in children. A comprehensive obesity prevention system involving government leadership, social participation, and collaboration between schools, families, and communities should be established. Additionally, health policies should target the dietary and lifestyle behaviors that contribute to childhood obesity. Such policies must be implemented with effective supervision, analysis, and evaluation to ensure the reduction of childhood obesity risk factors and lower the prevalence of childhood obesity.

## 5 Conclusion

In conclusion, low dietary diversity, nighttime snacking and excessive screen time are key risk factors for obesity among preschool children in this study area. To curb the growing prevalence of childhood overweight and obesity, comprehensive interventions should focus on optimizing children’s dietary structure, standardizing eating behaviors and reducing sedentary activities. Parents and kindergartens should cooperate to foster healthy living habits in young children. Further longitudinal research is warranted to confirm causal links and provide stronger evidence for obesity prevention initiatives.

## Data Availability

The original contributions presented in the study are included in the article/supplementary material, further inquiries can be directed to the corresponding author.
